# Pareto in Prison

**DOI:** 10.1002/bsl.2716

**Published:** 2025-01-16

**Authors:** Mark A. Morgan, Joshua S. Long, Matthew W. Logan, Frank Benton

**Affiliations:** ^1^ Department of Criminal Justice and Security Studies University of Dayton Dayton Ohio USA; ^2^ School of Criminology and Justice Studies University of Massachusetts‐Lowell Lowell Massachusetts USA; ^3^ School of Criminal Justice and Criminology Texas State University San Marcos Texas USA

**Keywords:** corrections, criminal behavior, officer safety, recidivism, rehabilitation, risk assessment

## Abstract

The Pareto principle is based on the concept that roughly 80% of outcomes are generated by 20% of inputs, efforts, or contributors within a group. Using a national sample of U.S. prison inmates, we examined various percentile rankings of self‐reported institutional misconduct to determine how much disorder is created behind bars by the most prolific offenders. Findings revealed that, regardless of sex, the top 20% of inmates were responsible for approximately 90% of all rule violations and write‐ups received. These general patterns remained similar even after adjusting infractions for time served in prison. Further analyses indicated that membership within these high‐rate groups was often significantly predicted by those who were younger, black, had more extensive criminal histories, committed violent crimes, resided in state facilities, anticipated being released, used drugs prior to their arrest, were diagnosed with a personality disorder or ADHD, and exhibited worse negative affect. Some sex‐specific effects were also observed. The disproportionate impact these chronic offenders have on the prison environment is detrimental to all individuals who live and work around them. Future research should investigate specific types of misconduct, distinct time intervals of incarceration, and facility effects such as management style, security levels, or offender composition.

## 1 Introduction

The Pareto principle, often referred to as the “80/20” rule, suggests that a disproportionate number of outcomes, typically about 80%, result from a relatively small number of inputs, usually around 20% (Juran 1954). Its application in the social sciences is widespread, offering explanations for the uneven distribution of wealth across social and economic strata, as well as the prevalence of specific behaviors and practices among various constituencies such as in commercial business or industry (Grosfeld‐Nir, Ronen, and Kozlovsky 2007). As a heuristic within the criminological literature, its logic—like variations on a theme—has been used to demonstrate the degree to which criminal events tend to cluster across multiple units of analysis (Caspi et al. 2016; A. R. Piquero, Farrington, and Blumstein 2003; Sherman, Gartin, and Buerger 1989; Vaughn et al. 2014; Wilcox and Eck 2011; Wolfgang, Figlio, and Sellin 1972; Zimring 1981). The observations made in these studies have been highly influential in the formation of criminal justice policy systemwide and span the fields of policing (Corsaro 2018), courts and sentencing (Bensen 2013), and correctional rehabilitation or reentry via offender classification (Bonta and Andrews 2016; Lowenkamp and Latessa 2005).

Although there exists a spate of research documenting the patterns and trajectories of antisocial behavior among offender populations and the locations at which their transgressions occur, comparatively fewer studies have evaluated the veracity of Pareto's principle *within* prisons (but see DeLisi 2003; Duwe 2020; Logan et al. 2023). This knowledge gap is noteworthy for several reasons. Chief among them is the fact that many career criminals go on to serve lengthy prison sentences for serious offenses, where they subsequently import many of the same risk factors that spurred their initial (and repeated) contact with the justice system (DeLisi et al. 2011; Walters and Crawford 2013). To this end, prisons are dangerous places in which to live and work. By virtue of the criminal histories these individuals import, they represent a tangible threat to the occupational mandate of those employed in prisons, where order maintenance and security are paramount to ensuring safety for staff and inmates alike (Sorensen et al. 2011; Steiner 2008). Moreover, while the prevalence of extremely violent incidents in correctional facilities is low, it is estimated that frontline correctional workers are still a staggering 36 times more likely to be injured due to violence relative to other professions (Konda et al. 2013). This concern is further evidenced by thousands of inmate injuries and deaths, over 100 correctional officer fatalities, and more than 125,000 non‐fatal employee injuries that have occurred in the U.S. state and federal prison systems over the past two decades (Carson and Cowhig 2020; Wolff and Shi 2009). Given that assaults are often the leading cause for these incidents, evidence‐based assessment of problematic inmates is crucial.

Our focus here is on applying the Pareto principle to the study of institutional misconduct among a national sample of U.S. inmates with the goal of identifying the most chronic and prolific rule breakers, based on self‐reports. It is important to note that other assessments of chronic misconduct in prison, while still informative, may be limited because they rely on official administrative data or are affected by regional variation (Steiner and Wooldredge 2014). Therefore, we are primarily interested in (1) modeling the distribution of self‐reported misconduct amongst the wider inmate population to assess whether it comports with Pareto's principle and (2) ascertaining the legal and extralegal correlates that are predictive of membership into respective misconduct categories (e.g., the 80th percentile). Understanding the patterns of chronic prison misconduct and those who are most likely to commit it may facilitate the design and implementation of (1) equitable prevention strategies, (2) rehabilitative programming or reentry methods, (3) sentencing modifications, and (4) suitable risk assessments—all of which comport with the best practices in promoting staff and inmate safety (French and Gendreau 2006; Latessa, Johnson, and Koetzle 2020).

## 2 Applying Pareto to Criminology

The Pareto principle is informed by the observations of Vilfredo Pareto (1896), an Italian sociologist and economist whose seminal work, *Cours d'Économie Politique*, empirically demonstrated that roughly 80% of land in Italy was owned by 20% of the population. Mathematically, Pareto's observations are illustrative of a power law distribution for a particular set of parameters—one that emphasizes the “vital few” and “trivial many” and one which appears widely across the social and behavioral sciences (Juran 1954; Newman 2005). Within criminology, its utility is twofold. First, at the aggregate level, Pareto's logic has been used to demonstrate that the vast majority of criminal events are geographically concentrated into comparatively few locations, including facilities, “hot spots,” street segments, and even cities (see Weisburd et al. 2024 for a comprehensive review). Second, at the individual level, the principle has been employed to document the fact that a small, but very active, proportion of offenders commit most of the crime observed in society. For example, Wolfgang, Figlio, and Sellin (1972) research on career criminals showed that approximately 6% of the study's males accounted for over 50% of all offenses—and a much larger share of serious violent crimes. Moffitt (1993) would later build on this work and propose a developmental taxonomy of offending to identify and differentiate life‐course‐persistent offenders—those who engage in a sustained pattern of antisocial behavior throughout their entire lives—from the more “normative” adolescence‐limited offenders. This line of inquiry later culminated in the Dunedin longitudinal study where 22% of the cohort was found to be responsible for 81% of the crime and a substantial percentage of associated “economic burden” (Caspi et al. 2016). The observations made and conclusions drawn by Wolfgang, Figlio, and Sellin (1972) and Moffitt (1993) have since served as a lynch pin for life‐course criminologists who have subsequently replicated their work across time and space, among both juvenile and adult populations (Falk et al. 2014; Forsyth et al. 2014; A. R. Piquero, Jennings, and Barnes 2012; Wright, Tibbetts, and Daigle 2014).

Given its utility, it is surprising that the Pareto principle has been sparsely applied to the study of prisons. Indeed, only a handful of studies have directly examined the determinants of offense prevalence across incarcerated groups (Butler et al. 2021; DeLisi 2003; Logan et al. 2023; Trulson et al. 2010). Collectively, this research reinforces three important points. First, they validate the notion that antisocial behavior (in the form of institutional misconduct) is highly concentrated among a relatively small group of individuals. For example, DeLisi's (2003) research, based on the official records of a sample of adults housed in a southwestern U.S. correctional facility, showed that fewer than 8% of inmates accounted for 100% of homicides, 75% of rapes, 80% of arsons, and 50% of aggravated assaults that occurred while in prison. Similar results were reported by Butler et al. (2021), only the asymmetric patterns of misconduct they observed were for delinquents who were committed to state juvenile correctional facilities in Texas (see also Trulson et al. 2010, 2022). Second, they corroborate arguments regarding the generality of deviance that are emblematic of career criminals for both males and females alike (Dembo et al. 1992; Hirschi and Gottfredson 1994; see also Smith, Cullen, and Latessa 2009). Most recently, Logan et al. (2023) found that the predictors of serious, chronic, and violent prison misconduct among high‐rate offenders were largely equivalent between men and women. Third and relatedly, each study converges on the fact that some of the most consistent predictors of repeat offending during incarceration are prior criminality, age, and race—all of which are congruent with the well‐known importation theory of inmate (mal)adjustment, which places a premium on the effects of pre‐prison characteristics to explain variation across institutional outcomes (Cao, Zhao, and Van Dine 1997; DeLisi, Berg, and Hochstetler 2004; see also Irwin and Cressey 1962).

The value of applying Pareto's concepts to the study of prisons is further supplemented by case studies of especially problematic inmates, the descriptions of whom illustrate just how much damage the “vital few” are capable of causing. Consider, for example, the case of “Mr. Z,” whose life history (including his extensive criminal career) was documented by DeLisi, Drury, and Elbert (2021). Mr. Z was a prolific offender since the age of 8, a time during which he exhibited major conduct disorders marked by frustration, aggression, rage, and feelings of invincibility. He was arrested for burglary and subsequently spent much of his life in and out of prison for serious violent felonies, including multiple counts of physical assault and armed robbery. These behavioral patterns continued and intensified during his incarceration, where he spent decades in administrative segregation and was part of a security threat group. During his interview, he confessed to killing at least 10 people in prison and was suspected of being involved in 13 other murders. Legal affidavits would later reveal that dozens of inmates expressed fear at the prospect of being victimized by him as a result of his notoriety and reputation. When prompted to explain his motives, Mr. Z showed no emotion and seemed to indicate that he was merely “[taking] care of business” for his prison gang (DeLisi, Drury, and Elbert 2021, 4). He was later assessed as being “floridly psychopathic,” having met the diagnostic criteria for antisocial personality disorder, amongst a host of other mental illnesses and dependencies. In total, Mr. Z was arrested approximately 50 times in the community and reprimanded for nearly 100 violations during his confinement (M. DeLisi, personal communication, August 19, 2024).

By the same token, Thomas Silverstein was a life‐long offender from a young age. He engaged in criminal behavior with the guidance of his mother and father and eventually received a 15‐year federal prison sentence for armed robbery. During his incarceration, staff promptly labeled him a “management problem…. displaying a predatory and assaultive behavior pattern” (Earley 2023, 56). He was later found guilty of the murders of three other prisoners, leading to the nickname “Terrible Tom” (Earley 1992). Infamously, on October 22, 1983, Silverstein was being escorted by three correctional officers inside USP Marion after a shower. He was shackled with restraints but, with the assistance of another inmate, was able to surreptitiously free himself whereby he then proceeded to stab one of the officers, Merle E. Clutts, to death. Just hours later, another officer, Robert L. Hoffmann, Sr., was slain by Silverstein's associate and “apprentice,” Clayton Fountain, in a different part of the prison. Allegedly, Fountain did not want Silverstein to beat his “score” in the number of people the two men had murdered. Their actions caused USP Marion to go into a complete lockdown—one that was not lifted for 23 years and which ushered in the “Marionization” movement of supermax prison development (Peters 2013). The transgressions of Silverstein and Fountain, as well as those of Mr. Z, underscore how the actions of a few individuals can have dire consequences for the rest of the prison population.

## 3 The Current Study

The current study is situated at the intersection of prison policy and offender classification to promote institutional safety for those who live and work in correctional facilities. Our aim is to identify those offenders that command the most attention in terms of time, resources, and threats to security. It is important to note that in the context of prisons, security concerns are varied and do not necessarily focus solely on violent offending due to the generality of deviance—and the reality that minor acts of misconduct (e.g., possessing contraband) can lead to or facilitate more serious acts of violent behavior (Goulette et al. 2022). Thus, while many studies have underscored the importance of controlling particularly aggressive or dangerous inmates (Van Der Vorst et al. 2024), such as Mr. Z, it is equally important to focus on the sheer quantity or frequency of offending even if non‐violent. Accordingly, we are principally interested in answering the following research questions, which are reflective of the extant literature on chronic offending and correspond with ongoing efforts to improve the welfare of all individuals within the prison environment:

RQ1: Does the Pareto principle apply to institutional misconduct in prison?

RQ2: What predicts classification into high‐rate risk groups of chronic misconduct?

RQ3: Are these predictors similar across sex and comparable to previous assessments?

## 4 Methods

### 4.1 Data Source and Sample

Our analyses are based on self‐report data obtained from the *Survey of Prison Inmates, United States, 2016* (ICPSR 37692) compiled by the Bureau of Justice Statistics (BJS 2024). This is a public‐use data set with face‐to‐face interview data collected from a nationally representative sample of persons 18 and older incarcerated in state and federal facilities across the United States in 2016. The prisons were first randomly selected from a list of 2001 unique facilities stratified by sex, jurisdiction, and self‐representing states (i.e., states that housed 100,000 or more prisoners), producing an initial selection of 385 facilities using probability proportionate to size of which 364 ultimately participated. During the second stage, a stratified random sample of 37,058 prisoners was selected from all eligible inmates confined within those facilities, yielding response rates of 69.3% for state prisoners and 72.8% for federal prisoners across the country. The data includes a total of 24,848 offenders who participated—20,064 state and 4784 federal—across 306 state and 58 federal prisons. The survey contains background information on inmate demographics, their criminal history, substance abuse issues, physical disabilities, mental health disorders, and rule violations for misconduct committed during confinement. Further information regarding the survey methodology can be acquired within the publicly accessible codebook or the BJS summary report (Glaze 2019).

### 4.2 Dependent Variable

The dependent variable for this study, *Misconduct*, is the total number of infractions the offender reported receiving since their current admission to prison. This includes the number of times the offender was written up for breaking any prison rules or for violations of which they were later found guilty. Overall, this measure ranges from 0 to 500 infractions with an average of 5.87, a median of 1.00, and a standard deviation of 22.44 for the full sample. We subsequently group these infractions in our analyses based on the offender's sex and their percentile ranking of total misconduct.

### 4.3 Independent Variables

#### 4.3.1 Justice System Attributes

*Federal Custody* indicates what type of agency the offender was being “held for” during their survey interview (0 = State, 1 = Federal).^1^ The state designation includes state correctional authorities or departments of correction and local correctional authorities such as jails or detention centers, while the federal designation includes the Federal Bureau of Prisons, U.S. Immigration and Customs Enforcement, and the U.S. Marshals Service. We apply three criminal history indicators to capture not just the persistence of offending, but also its onset and potential severity (DeLisi 2006). *First Arrest* indicates the earliest age at which the subject was first arrested for any offense. *Prior Arrests* is a continuous measure of how many times the respondent had been arrested in their entire life for any offense, including incidents that did not result in a court appearance or a conviction. Likewise, *Prison Terms* captures the number of times the offender had ever been sentenced to serve time in a state or federal prison including their current admission.^2^ *Anticipates Release* measures whether the offender expected having a definite date on which they were expected to be released from prison in the future (0 = No, 1 = Yes). *Years Served* denotes how long the subject had been in prison for their current offense, in years, and was calculated by subtracting their year of admission from the date of the survey.^3^

Given that dangerous and other “extreme” criminals are known to exhibit more prolific offense histories (DeLisi 2001), the offender's controlling offense which led to their current incarceration was classified, respectively, as either a *Violent Crime* (the reference category), a *Property Crime*, a *Drug Crime*, or a *Public Order Crime* (0 = No, 1 = Yes).^4^ *Prison Programs* is a count measure of up to three potential types of institutional programming the offender had actively engaged in since their admission to prison. These included ever participating in job training programs such as employee readiness or vocational training; ever taking educational programs such as high school/GED courses, adult basic education, college courses, or English as a second language classes; and, lastly, if the offender currently held a work assignment at the time of the survey either inside or outside the prison facility.

#### 4.3.2 Personal Characteristics

The respondent answered several demographic questions that are used as control variables including their *Age* at the time of the survey measured in years. Their race/ethnicity was captured through five mutually exclusive groupings of *White* (the reference category), *Black*, *Hispanic*, *Other Race*, or *Multiracial* (0 = No, 1 = Yes). *U.S. Citizen* signifies whether the subject was a U.S. citizen or not (0 = No, 1 = Yes). *Married* denotes whether the offender was currently married at the time of their interview as opposed to being divorced, widowed, or separated (0 = Not married, 1 = Married). *Children* indicates whether the subject had any biological or adopted children (0 = No, 1 = Yes). *Welfare* is a retrospective socioeconomic indicator that captures whether the offender's parents or guardians had ever received public assistance from the government before the subject turned 18 (0 = No, 1 = Yes). *Veteran* indicates if the subject had ever served in the U.S. Armed Forces (0 = No, 1 = Yes). *Education* captures how many years of formal schooling the respondent had received prior to their incarceration.^5^ *Prior Employment* indicates if the subject had worked in a job or at a business, excluding any illegal activities, in the 30 days before their arrest (0 = No, 1 = Yes).

*Alcohol Dependency* is categorized as a disorder of dependency or abuse as derived by BJS staff from a battery of 21 questions regarding the subject's problematic alcohol habits within the year prior to their arrest (0 = No, 1 = Yes). *Drug Use* was measured dichotomously and included seven possible drugs that the respondent could have reported using within the 30 days prior to their arrest: marijuana, cocaine, heroin, hallucinogens, methamphetamine, inhalants, or prescription drugs without a doctor's approval (0 = No, 1 = Yes). Health‐related indicators were also used given that these conditions are associated with inmate misconduct and can impair functioning or mobility within the prison environment (Grosholz and Semenza 2018; see also Salas‐Wright and Vaughn 2016; Travis, Western, and Redburn 2014). *Disability* is a dichotomous variable capturing any six possible indicators detailing physical or cognitive impairments the offender could have reported while incarcerated. These include being deaf or having difficulty hearing; being blind or having difficulty seeing; having difficulty concentrating, remembering, or making decisions due to a physical, mental, or emotional problem; having serious difficulty walking or climbing stairs; and having difficulty doing activities alone such as going to meal time, working, or attending classes and programming (0 = No, 1 = Yes). *Chronic Condition* is a dichotomous variable capturing any 11 possible health‐related problems or diseases the offender currently had or suffered from as diagnosed by a medical professional. These include cancer; hypertension; stroke; diabetes; heart disease or other heart problems; kidney problems; arthritis, gout, lupus, or fibromyalgia; asthma; cirrhosis of the liver; hepatitis B; and hepatitis C (0 = No, 1 = Yes). Body mass index (*BMI*) was calculated by standard formula using the subject's reported height and weight.

Seven separate mental disorders are captured dichotomously and indicate if the subject had ever reported being diagnosed with that condition by a mental health professional, such as a psychiatrist or psychologist, whether before or during their current incarceration (0 = No, 1 = Yes): *Bipolar* disorder (including manic depression or mania), *Depression*, *Schizophrenia* (including psychotic disorders), post‐traumatic stress disorder (*PTSD*), *Anxiety* (including panic disorders or obsessive‐compulsive disorder), *Personality* disorder (including antisocial or borderline personality), or an attention deficit disorder (*ADHD*). Lastly, *Negative Affect* is a summed composite measure of six individual variables capturing the amount of psychological stress, despondency, and maladjustment experienced by the respondent within the last 30 days prior to the survey (*α* = 0.820). The original six items include the reported frequency of nervousness, hopelessness, restlessness, depression, exhaustion, and worthlessness felt by the offender with each measured on an ordinal scale (0 = None of the time, 1 = A little of the time, 2 = Some of the time, 3 = Most of the time, 4 = All of the time). Given that the overall percentage of missing data per variable was low, we excluded cases using listwise deletion. Collinearity diagnostics reported an average variance inflation factor of 1.37 with no individual value exceeding 2.45 which is within the acceptable range for the social sciences (Fox 2016).

## 5 Results

**FIGURE 1:**
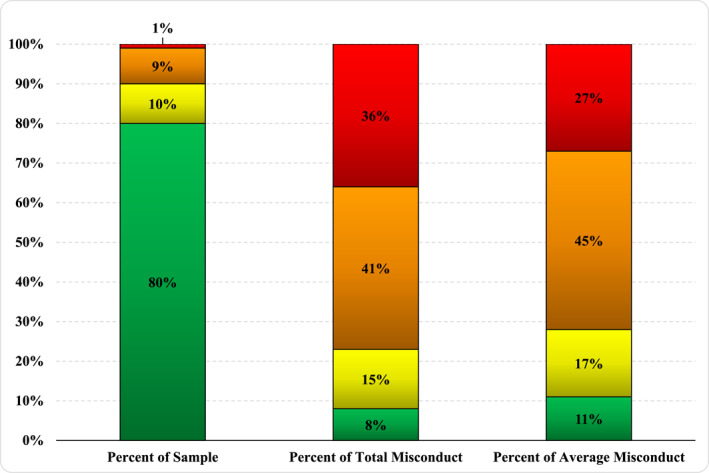
Percentage of misconduct committed by exclusive groups of females. For reference, the top 1% of female offenders in the sample committed 36% of all misconduct and 27% of all misconduct averaged by years served in prison.

**FIGURE 2:**
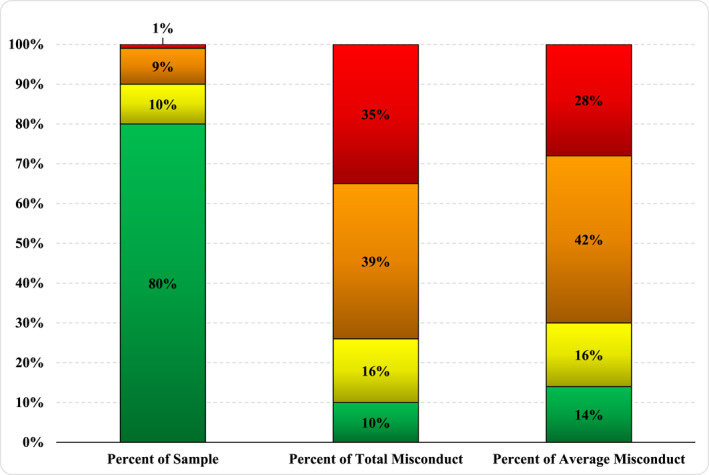
Percentage of misconduct committed by exclusive groups of males. For reference, the top 1% of male offenders in the sample committed 35% of all misconduct and 28% of all misconduct averaged by years served in prison.

Table 1 presents the descriptives statistics for each variable in the study categorized by sex as our research strategy involves within‐group analyses. As shown in Table 2, we next established thresholds for the 80th, 90th, and 99th percentiles of misconduct by examining the cumulative percentages of the total number of infractions among females and males, separately. For females, the threshold for the 80th percentile was three violations, eight for the 90th percentile, and 52 for the 99th percentile. A total of 66 female offenders were within the highest category and they were responsible for 36% (8986 write‐ups) of all incidents among women. Likewise, for males, five violations were required for the 80th percentile, 14 for the 90th, and 100 for the 99th percentile. A total of 259 males fell into the highest category and they produced 35% (41,180 write‐ups) of all reported misconduct among men. For a graphical depiction of the outsized influence that these high‐rate offenders have in the sample, Figures 1 and 2 showcase the percentage of total offenses committed by each percentile grouping, for females and males, respectively. Additionally, average misconduct, adjusted by the number of years spent in prison, is also presented in the third column.^6^ As expected, when time served is taken into account, this naturally deflates the impact of those in the 99th percentile, yet the general risk pattern does not deviate substantially. In summary, just 20% of the inmate sample, across both sexes, is responsible for approximately 90% of all self‐reported prison misconduct.

**TABLE 1:** Descriptive statistics.

	Females (*n* = 6307)			Males (*n* = 18,541)		
	Mean	SD	Range	Mean	SD	Range
Justice system attributes						
Misconduct	4.03	17.56	0–500	6.50	23.85	0–500
Federal custody	0.17	0.37	0–1	0.19	0.39	0–1
First arrest	23.94	10.80	6–74	19.51	9.38	6–81
Prior arrests	7.32	13.10	0–200	8.58	13.39	0–200
Prison terms	1.64	1.80	1–30	2.04	1.93	1–30
Anticipates release	0.78	0.40	0–1	0.76	0.42	0–1
Years served	2.94	4.55	0–44	5.63	7.11	0–48
Violent crime	0.32	—	0–1	0.49	—	0–1
Property crime	0.26	0.44	0–1	0.13	0.33	0–1
Drug crime	0.27	0.44	0–1	0.19	0.39	0–1
Public order crime	0.13	0.33	0–1	0.17	0.38	0–1
Prison programs	1.53	0.95	0–3	1.41	0.97	0–3
Personal characteristics						
Age	38.00	10.84	18–78	39.45	12.22	18–86
White	0.49	—	0–1	0.28	—	0–1
Black	0.18	0.39	0–1	0.34	0.47	0–1
Hispanic	0.15	0.36	0–1	0.24	0.42	0–1
Other race	0.02	0.15	0–1	0.02	0.15	0–1
Multiracial	0.13	0.33	0–1	0.10	0.30	0–1
U.S. Citizen	0.97	0.16	0–1	0.91	0.28	0–1
Married	0.17	0.37	0–1	0.15	0.35	0–1
Children	0.80	0.39	0–1	0.69	0.46	0–1
Welfare	0.40	0.49	0–1	0.40	0.49	0–1
Veteran	0.01	0.13	0–1	0.07	0.27	0–1
Education	11.74	2.59	1–18	11.05	2.51	1–18
Prior employment	0.49	0.50	0–1	0.62	0.48	0–1
Alcohol dependency	0.18	0.38	0–1	0.19	0.39	0–1
Drug use	0.64	0.47	0–1	0.61	0.48	0–1
Disability	0.47	0.49	0–1	0.37	0.48	0–1
Chronic condition	0.54	0.49	0–1	0.41	0.49	0–1
BMI	29.50	6.25	12.29–80.76	28.08	4.76	13.68–116.08
Bipolar	0.39	0.48	0–1	0.19	0.39	0–1
Depression	0.47	0.49	0–1	0.23	0.42	0–1
Schizophrenia	0.09	0.28	0–1	0.07	0.26	0–1
PTSD	0.34	0.47	0–1	0.11	0.31	0–1
Anxiety	0.45	0.49	0–1	0.17	0.38	0–1
Personality	0.16	0.37	0–1	0.09	0.29	0–1
ADHD	0.24	0.43	0–1	0.23	0.42	0–1
Negative affect	7.44	5.42	0–24	5.87	5.32	0–24

**TABLE 2:** Classification of the 80th, 90th, and 99th percentiles of misconduct.

	Females (*n* = 6196)				Males (*n* = 18,139)			
	Threshold^a^	Number of individuals	Number of infractions	Percent of infractions (24,998) (%)	Threshold^a^	Number of individuals	Number of infractions	Percent of infractions (117,960) (%)
80th percentile	3	1495	23,012	92	5	4265	105,538	90
90th percentile	8	634	19,337	77	14	1836	86,852	74
99th percentile	52	66	8986	36	100	259	41,180	35

^a^ Number of reported write‐ups necessary to qualify for this category.

The results of the logistic regression models shown in Table 3 identified risk factors that were significantly associated with an increased likelihood that a prisoner will fall into the top 1% of rule violators.^7^ For both females and males, a greater number of prior arrests, years served, and being black predicted membership. Furthermore, women who had ADHD were three times more likely to be in the top 1%. However, women who committed drug crimes, were U.S. citizens, and who grew up receiving welfare were significantly less likely to belong. Among men, residing in a state prison, reporting a younger age at first arrest, being younger, anticipating release, not participating in prison programs, not having an alcohol dependency, and being diagnosed with a personality disorder were significantly associated with membership.

**TABLE 3:** Logistic regression model for the 99th percentile of misconduct.

	Females (*n* = 5008)			Males (*n* = 14,592)		
	OR	95% CI	SE	OR	95% CI	SE
Justice system attributes						
Federal custody	0.61	0.13–2.79	0.47	0.19**	0.05–0.62	0.11
First arrest	0.93†	0.87–1.00	0.03	0.93**	0.89–0.97	0.02
Prior arrests	1.02**	1.00–1.03	0.00	1.01**	1.00–1.02	0.00
Prison terms	0.84	0.56–1.25	0.17	1.01	0.93–1.10	0.04
Anticipates release	0.96	0.49–1.89	0.33	1.82**	1.26–2.64	0.34
Years served	1.19***	1.12–1.27	0.37	1.21***	1.18–1.25	0.01
Property crime	0.61	0.24–1.55	0.29	0.95	0.57–1.59	0.25
Drug crime	0.24*	0.06–0.90	0.16	0.85	0.46–1.56	0.26
Public order crime	0.30	0.06–1.39	0.23	0.65	0.33–1.29	0.22
Prison programs	1.34	0.96–1.88	0.23	0.61***	0.52–0.72	0.05
Personal characteristics						
Age	0.94†	0.89–1.00	0.02	0.91***	0.88–0.93	0.01
Black	2.49*	1.18–5.25	0.94	1.65*	1.07–2.52	0.35
Hispanic	2.10	0.86–5.12	0.95	1.00	0.58–1.72	0.27
Other race	—	—	—	2.12	0.78–5.75	1.07
Multiracial	0.88	0.31–2.48	0.46	1.28	0.74–2.21	0.35
U.S. Citizen	0.20*	0.04–0.93	0.15	2.49	0.57–10.83	1.86
Married	1.04	0.40–2.70	0.50	0.58†	0.32–1.06	0.17
Children	0.83	0.44–1.59	0.27	1.22	0.89–1.67	0.19
Welfare	0.51*	0.27–0.93	0.15	1.23	0.90–1.69	0.19
Veteran	—	—	—	0.74	0.35–1.55	0.27
Education	0.91	0.79–1.04	0.06	0.92†	0.86–1.00	0.03
Prior employment	0.63	0.34–1.17	0.19	0.92	0.68–1.25	0.14
Alcohol dependency	0.76	0.36–1.56	0.28	0.65*	0.44–0.96	0.13
Drug use	2.08†	0.98–4.41	0.79	1.11	0.77–1.60	0.20
Disability	0.67	0.35–1.27	0.21	1.03	0.74–1.45	0.17
Chronic condition	0.95	0.49–1.84	0.31	1.16	0.84–1.61	0.19
BMI	0.95†	0.90–1.00	0.02	1.00	0.97–1.03	0.01
Bipolar	1.41	0.65–3.05	0.55	1.52†	0.97–2.38	0.34
Depression	0.84	0.41–1.71	0.30	0.81	0.52–1.27	0.18
Schizophrenia	1.04	0.40–2.73	0.51	0.79	0.47–1.32	0.20
PTSD	0.87	0.43–1.75	0.31	1.51†	0.95–2.40	0.35
Anxiety	0.90	0.44–1.86	0.33	1.20	0.76–1.88	0.27
Personality	1.86†	0.92–3.76	0.66	1.63*	1.06–2.51	0.35
ADHD	3.04**	1.55–5.99	1.05	0.78	0.53–1.15	0.15
Negative affect	1.04	0.98–1.10	0.03	1.01	0.97–1.04	0.01
Model *χ* ^2^			203.40***			499.49***
McFadden's *R* ^2^			0.32			0.23
Correctly classified			98.78%			98.63%

† *p* < 0.10, *p < 0.05, **p < 0.01, ***p < 0.001.

Significant risk factors for being in the top 10% of rule violators are shown in Table 4. Given the larger sub‐samples involved, more factors were found to be predictive of membership in this category than for the top 1%. For both females and males, being in a state prison, younger age at first arrest, more prior arrests, anticipating release, years served, committing a violent crime (as opposed to a drug or public order crime), younger age, being black, having used drugs before their arrest, being diagnosed with a personality disorder, and worse negative affect all significantly predicted membership. Three other significant predictors of note for women included being Hispanic or multiracial and bipolar disorder. For men, committing a property crime, participating in prison programs, having children, and being physically larger were significantly associated with a decreased risk, whereas growing up on welfare and having ADHD were positively associated with membership in the top 10% of rule violators.

**TABLE 4:** Logistic regression model for the 90th percentile of misconduct.

	Females (*n* = 5233)			Males (*n* = 14,592)		
	OR	95% CI	SE	OR	95% CI	SE
Justice system attributes						
Federal custody	0.28***	0.16–0.47	0.07	0.42***	0.31–0.57	0.06
First arrest	0.95***	0.93–0.97	0.00	0.95***	0.93–0.96	0.00
Prior arrests	1.01**	1.00–1.01	0.00	1.01***	1.00–1.01	0.00
Prison terms	0.92	0.83–1.01	0.04	1.02	0.98–1.06	0.01
Anticipates release	1.31*	1.01–1.70	0.17	1.43***	1.22–1.67	0.11
Years served	1.22***	1.19–1.25	0.01	1.22***	1.21–1.24	0.00
Property crime	0.75†	0.56–1.01	0.11	0.81*	0.65–0.99	0.08
Drug crime	0.45***	0.33–0.63	0.07	0.55***	0.43–0.71	0.06
Public order crime	0.52**	0.34–0.80	0.11	0.59***	0.46–0.75	0.07
Prison programs	1.01	0.90–1.14	0.06	0.80***	0.74–0.86	0.02
Personal characteristics						
Age	0.95***	0.93–0.97	0.00	0.91***	0.90–0.92	0.00
Black	1.70***	1.27–2.27	0.25	1.32**	1.11–1.57	0.11
Hispanic	1.95***	1.43–2.67	0.31	1.09	0.89–1.35	0.11
Other race	1.76†	0.90–3.42	0.59	1.37	0.87–2.14	0.31
Multiracial	1.56**	1.14–2.13	0.24	1.21†	0.97–1.52	0.13
U.S. Citizen	0.89	0.37–2.14	0.39	1.26	0.84–1.90	0.26
Married	0.87	0.62–1.20	0.14	0.83	0.66–1.04	0.09
Children	0.98	0.76–1.26	0.12	0.83*	0.73–0.95	0.05
Welfare	1.11	0.89–1.38	0.12	1.19**	1.04–1.36	0.08
Veteran	0.74	0.30–1.82	0.34	1.03	0.76–1.38	0.15
Education	0.97	0.93–1.02	0.02	0.98	0.95–1.02	0.01
Prior employment	0.88	0.70–1.09	0.09	0.92	0.81–1.05	0.06
Alcohol dependency	1.05	0.81–1.35	0.13	0.90	0.77–1.06	0.07
Drug use	1.85***	1.41–2.44	0.25	1.58***	1.35–1.85	0.12
Disability	1.04	0.82–1.32	0.12	0.89	0.77–1.03	0.06
Chronic condition	0.92	0.73–1.16	0.10	1.14†	0.99–1.31	0.08
BMI	0.99	0.97–1.01	0.00	0.98*	0.97–0.99	0.00
Bipolar	1.33*	1.02–1.74	0.18	1.06	0.88–1.29	0.10
Depression	1.00	0.77–1.29	0.13	1.08	0.89–1.30	0.10
Schizophrenia	0.79	0.55–1.13	0.14	1.07	0.85–1.36	0.12
PTSD	0.90	0.70–1.16	0.11	0.96	0.78–1.19	0.10
Anxiety	0.89	0.69–1.15	0.11	1.14	0.94–1.39	0.11
Personality	1.49**	1.13–1.95	0.20	1.71***	1.40–2.09	0.17
ADHD	1.24†	0.97–1.58	0.15	1.31**	1.12–1.53	0.10
Negative affect	1.04***	1.02–1.06	0.01	1.01**	1.00–1.03	0.00
Model *χ* ^2^			951.07***			2871.04***
McFadden's *R* ^2^			0.27			0.29
Correctly classified			90.90%			90.55%

† *p* < 0.10, *p < 0.05, **p < 0.01, ***p < 0.001.

Lastly, to fully capture the essence of the Pareto principle, the characteristics of the top 20% of offenders were assessed in Table 5. For both females and males, residing in a state prison, younger age at first arrest, more prior arrests, anticipating release, years served, committing a violent offense (as opposed to all other types), younger age, being black, having been unemployed before their arrest, prior drug use, personality disorder, ADHD, and worse negative affect all significantly predicted membership. Notably, participation in prison programs was protective for males, but detrimental for females which may be an artifact of women exhibiting greater rehabilitative needs (Heilbrun et al. 2008). Among women, being Hispanic or multiracial and having a bipolar disorder once again significantly predicted membership. For males, a greater number of prior prison terms, U.S. citizenship, not having children, growing up on welfare, and reporting a chronic health condition were all significantly associated with belonging to the top 20% of offenders.

**TABLE 5:** Logistic regression model for the 80th percentile of misconduct.

	Females (*n* = 5233)			Males (*n* = 14,592)		
	OR	95% CI	SE	OR	95% CI	SE
Justice system attributes						
Federal custody	0.50***	0.38–0.65	0.06	0.47***	0.39–0.56	0.04
First arrest	0.97***	0.96–0.98	0.00	0.96***	0.95–0.97	0.00
Prior arrests	1.01***	1.00–1.02	0.00	1.00***	1.00–1.01	0.00
Prison terms	1.01	0.96–1.05	0.02	1.03*	1.00–1.06	0.01
Anticipates release	1.22*	1.01–1.47	0.11	1.22**	1.08–1.37	0.07
Years served	1.24***	1.21–1.27	0.01	1.22***	1.21–1.24	0.00
Property crime	0.72**	0.59–0.90	0.07	0.74***	0.63–0.86	0.05
Drug crime	0.63***	0.51–0.77	0.06	0.61***	0.53–0.71	0.04
Public order crime	0.62**	0.47–0.82	0.08	0.65***	0.56–0.76	0.05
Prison programs	1.19***	1.10–1.30	0.05	0.92**	0.88–0.97	0.02
Personal characteristics						
Age	0.95***	0.94–0.96	0.00	0.93***	0.92–0.93	0.00
Black	1.39**	1.12–1.73	0.15	1.25**	1.10–1.42	0.08
Hispanic	1.34*	1.07–1.69	0.15	1.13†	0.98–1.31	0.08
Other race	1.00	0.59–1.67	0.26	1.16	0.83–1.62	0.19
Multiracial	1.52***	1.22–1.89	0.17	1.01	0.85–1.20	0.08
U.S. Citizen	1.18	0.62–2.22	0.38	1.37*	1.04–1.78	0.18
Married	0.84	0.67–1.04	0.09	0.94	0.81–1.10	0.07
Children	0.85†	0.70–1.02	0.08	0.88*	0.80–0.98	0.04
Welfare	1.03	0.88–1.21	0.08	1.21***	1.10–1.34	0.06
Veteran	0.80	0.44–1.47	0.24	0.94	0.75–1.17	0.10
Education	0.98	0.95–1.02	0.01	0.98	0.96–1.00	0.01
Prior employment	0.85*	0.73–0.99	0.06	0.81***	0.74–0.90	0.04
Alcohol dependency	0.99	0.82–1.19	0.09	0.98	0.88–1.10	0.05
Drug use	1.61***	1.33–1.95	0.15	1.59***	1.43–1.78	0.09
Disability	0.98	0.82–1.16	0.08	1.03	0.92–1.14	0.05
Chronic condition	0.99	0.84–1.17	0.08	1.13*	1.01–1.25	0.06
BMI	0.99	0.98–1.00	0.00	0.99†	0.98–1.00	0.00
Bipolar	1.23*	1.02–1.48	0.11	1.12	0.96–1.29	0.08
Depression	1.04	0.87–1.25	0.09	1.04	0.91–1.20	0.07
Schizophrenia	1.07	0.82–1.38	0.13	1.01	0.84–1.22	0.09
PTSD	1.00	0.83–1.19	0.08	0.92	0.78–1.08	0.07
Anxiety	1.01	0.84–1.21	0.09	1.02	0.88–1.18	0.07
Personality	1.32**	1.08–1.62	0.13	1.25**	1.06–1.48	0.10
ADHD	1.30**	1.09–1.55	0.11	1.25***	1.11–1.40	0.07
Negative affect	1.03***	1.01–1.04	0.00	1.02***	1.01–1.03	0.00
Model *χ* ^2^			1317.16***			4705.51***
McFadden's *R* ^2^			0.22			0.29
Correctly classified			81.06%			82.76%

† *p* < 0.10, *p < 0.05, **p < 0.01, ***p < 0.001.

Taken together, the core risk factors for prolific misconduct comport with the broader scientific literature (Berk, Kriegler, and Baek 2006; Gendreau, Goggin, and Law 1997; Goetting and Howsen 1986; Steiner, Butler, and Ellison 2014) and center around criminal history measures such as the inmate's age at first arrest, their number of prior arrests, time served in prison, and the commission of a violent crime which led to their incarceration. Other relevant justice system attributes included anticipating a release date from prison, residing in a state facility, and the protective effect of program participation for men. Moreover, several sociodemographic variables were generally consistent across sex and percentile ranking including younger inmate age, black race, drug use prior to arrest, personality disorder, ADHD, and negative affect. While speculative, the dual effects of personality disorder and ADHD may represent a toxic interaction between psychopathic traits and low self‐control (DeLisi et al. 2018).^8^ Across sex there were few major differences, but specifically for women, Hispanic ethnicity or a multiracial background as well as bipolar disorder increased risk. On the other hand, for men, having children reduced their propensity while growing up impoverished materialized as a potential hazard.

## 6 Discussion

The results of the current study demonstrate that Pareto's principle is well‐suited to examining institutional misconduct—especially given the more extreme 90/20 ratio we observed. Indeed, this philosophy applies to rule violations in prison just as it does in the realms of business or economics. In those domains, the 20% that generate 80% of outcomes are typically those who produce the most goods, make the most sales, or contribute the most to successful ventures. In our study, the 20% refers to the prisoners who make life especially difficult for everyone else. This small cadre of prisoners are those who bother, bully, harass, and terrorize the rest of the prison population, and are akin to the “ball busters,” “gorillas,” “toughs,” and “wolves” once described by Gresham Sykes (1958). In fact, these “vital few” are so prolific in their offending that they justify enormous budgetary expenditures to control their behavior, including the construction of high‐security housing units. They are also the reason the Federal Bureau of Prisons needed to build Alcatraz, USP Marion, and ADX Florence and why other inmates are unable to “do their own time” in peace (Ward 2009). The “trivial many” (i.e., the remaining 80% of the prison population) live in fear that they will be confronted or victimized by the vital and dangerous few (DiIulio 1990; Irwin 1980, 2004). In recognizing the outlier influence of the top 20% of unruly prisoners, we necessarily acknowledge that the majority should not be lumped in with them, and that most prisoners self‐report committing few, if any, acts of misconduct during their incarceration.

This logic echoes prior comments made by Sherman (2007) in his discussion of the role that the “power few” play in criminological research and harm reduction strategies. His suggestions were based on the idea that available scientific resources should be reallocated, in large quantities, into the most harmful and homogenous tail of the offending distribution (i.e., those who are at the highest risk of committing serious criminal acts) to maximize favorable outcomes. In the context of the current study, this might translate into observable, appreciable reductions in institutional misconduct among certain inmates, particularly prolific and physically dangerous rule violators. Importantly, however, Sherman (2007) indicates that identifying such individuals is but a useful *starting point* for experimental interventions, as opposed to yielding definitive solutions given that the history of experimentation is replete with failure and iatrogenic effects. He remarks that the “power few” are not simply the “low‐hanging fruit,” but rather the “hardest nuts to crack”—the most difficult cases to solve and the most resistant to change—yet they are also those with the best chance of producing a big, successful result (308).

The collective body of research on the efficacy of rehabilitative programming as a harm reduction strategy among inmates is illustrative of this point. Recent, large‐scale evaluations based on randomized controlled trials suggest that psychological interventions in prison—such as cognitive behavioral therapy (CBT)—yield little to no substantial effect on the cessation of future antisocial behavior (Beaudry et al. 2021; see also Fazel et al. 2024). This is especially vexing from a research perspective because these are the very individuals for whom Sherman's (2007) proposed strategy *should* be the most effective; namely, methodologically rigorous programs explicitly targeting those with “high base rates, high seriousness, and high proportions of the total harm in a much larger population” (309). At the same time, Sherman posited that programmatic failures and their attributing factors are of great value because they shed further light on “what doesn't work”—a phrase which has become an operational lodestar in the correctional rehabilitation literature (Latessa, Johnson, and Koetzle 2020). This unsavory reality begs a fundamental policy question: where should departments of correction, agencies that are often confronted with fiscal and budgetary limitations, invest their resources? The answer depends largely on the philosophy of punishment to which one subscribes and is beyond the scope of this paper to address; nonetheless, its consideration underscores the utility of using Pareto in prison.

Accordingly, we maintain that the primary strength of applying the Pareto principle to the study of prisons is in its practical applications for facility management; most notably, those efforts pertaining to classification policies and practices used by administrators to maximize the effectiveness of their security procedures. In Ramos v. Lamm (1979), the court ruled that “any systems of classification, placement and assignment must be clearly understandable, consistently applied and conceptually complete. Methods of validation must be implemented and means of redress for irregularity must be provided” (170). Since prison administrators are held responsible for their decisions, they need to rely on valid, verifiable risk assessment procedures rather than judgments that are without a strong evidence base (i.e., practices that are arbitrary or capricious) (Austin and McGinnis 2004; Belbot and del Carmen 1993; MacKenzie 1989). Any factors determined to be predictive of misconduct should be subject to continual evaluation over time, and statistical validity should be demonstrated for specific populations.

Indeed, classification is the “first step” into the prison system of each state, and therefore holds a prominent place in setting up prisoners for success or failure (Duwe 2020). Agencies should attempt to place prisoners in the “least restrictive” environment possible so they can exercise their autonomy, access rehabilitative programming and family visits, and reduce costs. These goals can only be achieved if state agencies and researchers continually evaluate classification decisions to improve their functioning. While our study revealed risk factors that overlap with traditional classification criteria, it also suggests that there are some dynamic individual factors that should be considered, including substance abuse, ADHD, bipolar or personality disorders, and negative affect. Similarly, these concerns align with Beaudry et al. (2021) optimistic yet circumscribed findings regarding the use of therapeutic communities directly targeting substance abuse—programs which were likewise shown to reduce institutional violence in another systematic review (Auty, Cope, and Liebling 2017; see also French and Gendreau 2006). Nevertheless, as Yoon, Slade, and Fazel (2017) remark, prison inmates exhibit a constellation of comorbidities, including psychopathy, substance abuse, and personality disorder amongst others, and “if research and treatment pathways fail to take these comorbidities into account, any treatment approach that focuses on a single diagnostic group may encounter difficulties” (790).

The key to proper classification, then, is perhaps not necessarily the initial placement decision, but instead the decision to transfer a rule violator to a higher security level. How many acts of misconduct are necessary to justify an upward transfer? How long does a prisoner need to refrain from misconduct before they can be safely transferred down to a lower security setting? Do the higher security settings merely house the worst rule violators in a single location? Would they be better served in a rehabilitative setting rather than a restrictive setting? The answers to these questions are nuanced and depend on a litany of factors, some of which we identified (Kigerl and Hamilton 2016; see Steiner and Wooldredge 2019 for a detailed explanation). Along these lines, some research suggests that a closer inspection of sex differences in disciplinary responses might also be warranted, especially given the more extensive histories of trauma and other comorbidities female offenders are likely to import (Houser and Belenko 2015; Lahm 2017). The results of the current study, however, cannot resolve the controversies over violence reduction policies or practices because the models cannot predict future behavior perfectly, but they do demonstrate the influence that high‐risk people have on the prison environment. Consequently, researchers should continue to focus on exploring the sociodemographic characteristics of the “vital few” who play the most critical role in prison disorder.

The outcomes and limitations of our study also offer several avenues for future research. Perhaps most importantly, while we demonstrated that misconduct is highly concentrated among certain subgroups of inmates, we were unable to differentiate the *type* of rule violations they committed or for which they were later found guilty. As mentioned, prisons are based on order maintenance and security, and the inability to parse out violent from non‐violent misconduct offers only basic insights into the offending profiles of these individuals (Kuanliang and Sorensen 2008). Another aspect, noted in the accompanying codebook, is that some facilities denied access to prisoners who were deemed to be “too violent to be interviewed” (BJS 2024, 12).^9^ Scholars should, therefore, be mindful of this distinction and analyze differences where feasible. Notwithstanding this constraint, it is possible (and probable) that many of the individuals who met the criteria for the top 20% of rule violators are indeed at least versatilely violent, as research on the generality of offending indicates (Brame et al. 2014; A. Piquero 2000; Pratt et al. 2016; Pratt and Turanovic 2019). These are also the individuals who, irrespective of their type of preferred misconduct, command the most attention from prison staff. Even if they are predominantly non‐violent inmates, the sheer volume of their offending presents an arguably greater threat to correctional personnel in terms of time and resources; an especially salient concern, given the unprecedented fiscal and staffing crises that currently plague the American correctional system (Logan, Adams, and Mastracci 2024; Schaufeli and Peeters 2000; Scott‐Hayward 2009; Spanoudakis et al. 2024).

Like other studies, we could not fully disentangle all pre‐prison behaviors and histories from in‐prison risk factors, and some key variables like gang membership went unmeasured (DeLisi, Berg, and Hochstetler 2004). As a result, we cannot conclusively establish whether the observed patterns are necessarily reflective of the importation or deprivation models of prison adjustment. Relatedly, the data on which our analyses are based is cross‐sectional, which prohibits us from assessing the trajectories of individuals over the course of their prison sentence. Thus, where possible, researchers should aim to conduct longitudinal assessments of persistent prison misconduct, as it would elucidate the degree to which offending careers are stable behind bars and have practical implications for offender classification and placement. Similarly, although we used data from a national sample, we were unable to account for geographic or managerial differences that may exist between correctional facilities, including their security levels, and the corresponding composition of their respective inmate populations (Wooldredge 2020). As such, future studies should attempt to disaggregate data to account for regional differences that can contribute to variation in prison misconduct (Steiner, Butler, and Ellison 2014; Wooldredge, Griffin, and Pratt 2001). That said, facility effects, which might be accounted for with clustering, could still remain difficult to accurately assess when misconduct is measured over an extended period of time (e.g., since admission) as an offender may be transferred within and across facilities during a lengthy prison sentence. Finally, while administrative data tends to underestimate inmate misconduct (Steiner and Wooldredge 2014), the self‐reports used in our study still refer to detected violations and would, therefore, benefit by efforts to complement such information with surveys of unobserved behaviors or even official records to provide further clarity on the nature and extent of chronic offending in prisons moving forward.

## Author Contributions

**Mark A. Morgan:** writing–review and editing, writing–original draft, methodology, formal analysis, data curation, conceptualization. **Joshua S. Long:** writing–review and editing, writing–original draft, methodology, formal analysis, data curation, conceptualization. **Matthew W. Logan:** writing–review and editing, writing–original draft, conceptualization. **Frank Benton:** writing–review & editing, conceptualization.

## Ethics Statement

The authors have nothing to report.

## Conflicts of Interest

The authors declare no conflicts of interest.

## Data Availability

The data that support the findings of this study are openly available in NACJD at https://doi.org/10.3886/ICPSR37692.v5, reference number ICPSR 37692.

## Endnotes

^1^
There were 28 cases who responded, “some other authority” and were excluded.

^2^
We capped *Prior Arrests* at 200 and *Prior Prison Terms* at 30, the 99.9th percentile for each measure, due to heavy skew which affected 30 cases in total.

^3^
We excluded one case who was listed as having been incarcerated for 101 years yet was only 28 years old.

^4^

*Public Order Crime* also includes 137 cases of “other” unspecified crimes.

^5^
There were 141 cases that indicated they attended school in another country that was not comparable to the U.S. system and were excluded.

^6^
Since recently admitted inmates had not yet served a full year at the time of the survey, years served were adjusted upward by one year for all cases to average this estimate.

^7^
Although we denote variables in our tables that met the *p* < 0.10 threshold for interested readers, we do not refer to these as significant findings in our results.

^8^
While low self‐control is generally viewed as conceptually distinct from ADHD, we had no measure available to capture low self‐control, so ADHD within our study may have served to tap into underlying behavioral impulsivity (Aguilar‐Cárceles and Farrington 2020).

^9^
Some inmates who were not interviewed or otherwise deemed ineligible to participate were unavailable due to work assignments, were hospitalized, were in restrictive housing for non‐medical reasons, were mentally incapable, or were physically unable to complete the survey.
